# Emergence of genetic diversity and multi-drug resistant *Clostridium perfringens* from wild birds

**DOI:** 10.1186/s12917-024-04168-8

**Published:** 2024-07-06

**Authors:** Xinglong Song, Zhenyu Zhong, Jiade Bai, Tianchun Pu, Xuehan Wang, Hongxuan He, Yaqian Chen, Congshan Yang, Qingxun Zhang

**Affiliations:** 1 College of Animal Science and Technology, Anhui Agricultural University, Hefei, China; 2 Beijing Milu Ecological Research Center, Beijing Academy of Science and Technology, Beijing, China; 3 Beijing Key Laboratory of Captive Wildlife Technologies in Beijing Zoo, Beijing, China; 4 School of Biomedicine, Beijing City University, Beijing, China; 5 National Research Center for Wildlife-Born Diseases, Institute of Zoology, Chinese Academy of Sciences, Beijing, China

**Keywords:** *Clostridium perfringens*, Prevalence, Antimicrobial resistance, Genetic diversity, Wildlife

## Abstract

**Background:**

*Clostridium perfringens (C. perfringens)* is an important zoonotic microorganism that can cause animal and human infections, however information about the prevalence status in wild birds of this pathogenic bacterium is currently limited.

**Result:**

In this study, 57 strains of *C. perfringens* were isolated from 328 fecal samples of wild birds. All the isolates were identified as type A and 70.18% of the isolates carried the *cpb2* gene. Antimicrobial susceptibility testing showed that and 22.80% of the isolates were classified as multidrug-resistant strains. The MLST analysis of the 57 isolates from wild birds was categorized into 55 different sequence types (STs) and clustered into eight clonal complexes (CCs) with an average of 20.1 alleles and the Simpson Diversity index (Ds) of 0.9812, and revealed a high level of genetic diversity within the *C. perfringens* populations. Interestingly, the isolates from swan goose were clustered in the same CC while isolates from other bird species were more scattered suggesting that a potential difference in genetic diversity among the *C. perfringens* populations associated with different bird species.

**Conclusion:**

*C. perfringens *exhibits a wide range of host adaptations, varying degrees of antimicrobial resistance, and a high degree of genetic diversity in wild birds. Understanding the prevalence, toxin type, antimicrobial resistance, and genetic diversity of *C. perfringens* in wildlife populations is essential for developing effective strategies for disease control and management.

## Introduction

*Clostridium perfringens* is a spore-forming and gram-positive anaerobic bacterium that could cause gas gangrene, food poisoning, and necrotizing enteritis in humans and animals [1, 2]. *C. perfringens* is classified into seven types (from A to G) according to the combination of the six typing toxins: α-toxin (CPA), β-toxin (CPB), ε-toxin (ETX), ι-toxin (ITX), enterotoxin (CPE), and NetB [3]. It also produces non-typing toxins such as β2-toxin (CPB2), BEC, and NetF that are considered to be associated with specific diseases [4]. The toxin-based typing methods are widely used to investigate the epidemiology, causes, and diagnosis of *C. perfringens* infections. However, this method has intrinsic defects in discriminating particular *C. perfringens* subtypes from distinctpathotypes [5, 6]. Multi-locus sequence typing (MLST) has been extensively applied as the gold standard method for bacteria typing and bacterial population genetics analysis [7]. MLST analysis was performed based on sequencing of eight housekeeping genes of *C. perfringens* for generating different sequence types and clonal complexes [8]. There are two main groups of housekeeping genes that have been used in the MLST analysis of *C. perfringens*. The first group of housekeeping genes is *plc*, *dut*, *glpK*, *gmk*, *sod*, *tpi*, *ddlA* and *recA* which were first used by Jost for *C. perfringens* in 2006 [8], and the other is the PubMLST database which uses *gyrB*, *sigK*, *sodA*, *groEL*, *pgk*, *nadA*, *colA*, and *plc*, and the former group of housekeeping genes is more widely used than the latter.

Over the past few decades, multidrug resistance strains of *C. perfringens* are increasingly reported in humans and animals which may be caused by the abuse of antibiotics [9, 10]. It has been reported that *C. perfringens* showed high percentages of resistance to tetracycline, erythromycin, lincomycin, neomycin, erythromycin, and sulfonamides in various types of animals around the world, and exhibited varying degrees of resistance to enrofloxacin, penicillin, fluoroquinolones, and doxycycline [11–14]. Recently, the novel plasmid-borne ABC transporter gene optrA identified from *C. perfringens* isolates of animal origin conferred combined resistance to antimicrobial agents in clinics including oxazolidinones and phenicols [15]. The increasing antibiotic resistance of *C. perfringens* in different animals should raise awareness of global public health security and their control strategy.

Wildlife serves as a potential reservoir for antibiotic-resistant bacteria, and there is a strong correlation between the resistant strains carried by wild birds and those found in human and animal isolates [16–18]. Some studies have shown that wild birds have the potential to transmit *C. perfringens* to poultry [18]. Antibiotic-resistant bacteria can be transmitted to humans through direct contact with infected wild birds or indirectly through exposure to polluted environmental components [19]. Although *C. perfringens* is widely present in humans, food, and livestock animals [20–23], there are still limited data on *C. perfringens* in wild birds and the threat to public health posed by wild birds carrying *C. perfringens* cannot be ignored. Here, we investigated the prevalence, toxin type, antimicrobial resistance patterns, and genetic diversity of *C. perfringens* from different wild birds in Beijing, China. This epidemiological investigation on *C. perfringens* in wild birds provided a reference for monitoring multidrug-resistant bacteria in wildlife.

## Material and method

### Sample collection and DNA extraction

A total of 328 fecal samples from Night heron (*Nycticorax nycticorax*), Grey heron (*Ardea cinerea*), Bar-headed geese (*Anser indicus*), Swan goose (*Anser cygnoides*), Mallard duck (*Anas platyrhynchos*), Indian peafowl (*Pavo cristatus*), Daurian jackdaw (*Corvus dauuricus*) were collected from different wetland park in Beijing, China, between 2022.12 and 2023.04 (winter and spring). Among them, grey herons, night herons, mallard duck and swan geese are migratory birds, while the others are non-migratory birds. Fecal samples were collected into sterile tubes and mixed with PBS buffer and then inoculated on selective Tryptose Sulfite Cycloserine (TSC) agar medium, the single colonies were picked and cultured in Fluid Thioglycollate Medium (FTG). Genomic DNA of cultured bacteria was extracted with TIANamp bacteria DNA kit (TIANGEN Biotech CO., LTD, Beijing) according to the manufacturer’s instructions.

### Toxin gene detection

Multiplex PCR was used to examine the presence of the *cpa*, *cpb*, *etx*, *iap*, *cpe*, *netB*, and *cpb2* genes from the isolates (Table 1) [24, 25]. Four reference strains including *C. perfringens* Type A (CVCC 2015), *C. perfringens* Type B (CVCC 54), *C. perfringens* Type C (CVCC 1153), and *C. perfringens* Type D (CVCC 60,201) from our previous studies were used as positive controls for toxin typing [26]. Electrophoresis was performed on a 1% agarose gel with Gel Red using standard procedures.

### Antimicrobial susceptibility test

The susceptibility of the *C. perfringens* isolates to 11 antimicrobial agents (Meropenem, tetracycline, ceftriaxone, penicillin, ampicillin, levofloxacin, piperacillin, erythromycin, Gentamycin, chloramphenicol, and lincomycin) was determined based on the broth micro dilution method suggested by the Clinical and Laboratory Standards Institute (CLSI) [27]. In brief, we employed a 96-well microplate covered with FTG, and each well was dispensed with 100 µL antibiotic and 100 µL *C. perfringens*. The plates were incubated at 37℃ for 24 h in an anaerobic atmosphere. The MIC values were defined as the lowest concentration that produces complete inhibition of *C. perfringens*. The susceptibility results were interpreted according to the CLSI 2019 guidelines [27].

### Sequencing of housekeeping genes

According to the *C. perfringens* MLST method established by Hibberd et al. and Jost et al [8, 28], eight housekeeping genes (*plc*、*dut*、*glpK*、*gmk*、*sod*、*tpi*、*ddlA*、*recA*) were selected for PCR amplification (Table 1). PCR assays were performed in the final volume of 50µL containing 25µL of 2×PCR Taq Mastermix; 1µL of each primer (10mmol/L); 1µL of DNA template, and 22µL double-distilled water. Reactions were performed with initial denaturation at 94 °C for 5 min, at 94 °C for 30s, at 55 °C for 60s and at 72 °C for 60s, followed by 35 cycles and a final elongation at 72 °C for 10 min. Then the PCR products were sequenced by Tsingke Biotechnology Co., Ltd. (Beijing, China), and the same primers were used for amplification and sequencing.

### MLST and evolutionary relationship analysis

The genetic relationship of 57 isolates of *C. perfringens* from wild birds were analyzed using MLST. The above-mentioned sequencing nucleotide sequences of the eight housekeeping genes were aligned and processed using BioNumerics 7.6 software to create an allele database, and sequence type (ST) was given to each strain. The minimum spanning tree was plotted using the minimum spanning tree method in BioNumerics software. This method is based on the allelic differences between different ST types, in which isolates with at least seven alleles being the same are defined as clone complex (CC), and both ST and CC are considered MLST subtypes [28]. Simpson’s diversity index (Ds) was also used to assess the genetic diversity of the isolates [29–31].

## Result

### Occurrence and toxin types of *C. perfringens*

A total of 57 strains of *C. perfringens* were isolated from the 328 fecal samples of wild birds with a recovery rate of 17.38%. The positive rates varied greatly in different hosts, ranging from 2.32% (Daurian jackdaw) to 54.76% (Grey heron) (Table 2). All isolates of different regions were identified as *C. perfringens* type A, which means that *cpb*, *etx*, *iap*, *cpe*, and *netB* toxin genes were not detected in all isolates. The high detection rate of the *cpb2* gene in all *C. perfringens* isolates was 70.18% (40/57) (Table 2).

**Table 1:** Prevalence and toxin gene profiles of *C. Perfringens* isolates from different host

Sample type		Host	Number of samples analyzed	Positive samples (%)	Toxin gene presence	
					cpa Positive samples (%)	cpa + cpb2 Positive samples (%)
Fecal		Night heron	60	21 (35.0)	7 (33.33)	14 (66.67)
Fecal		Grey heron	42	23 (54.76)	5 (21.74)	18 (78.26)
Fecal		Bar-headed geese	6	1 (16.67)	1 (100.0)	0 (0.0)
Fecal		Swan goose	97	6 (6.18)	1(16.67)	5 (83.33)
Fecal		Mallard duck	53	4 (7.55)	2(50.00)	2 (50.00)
Fecal		Indian peafowl	27	1 (3.70)	0 (0.0)	1(100.0)
Fecal		Daurian jackdaw	43	1 (2.32)	1 (100)	0 (0.0)
	Total		328	57 (17.38)	17 (29.82)	40 (70.18)

### Antibiotic resistance profiles

**Fig. 1:**
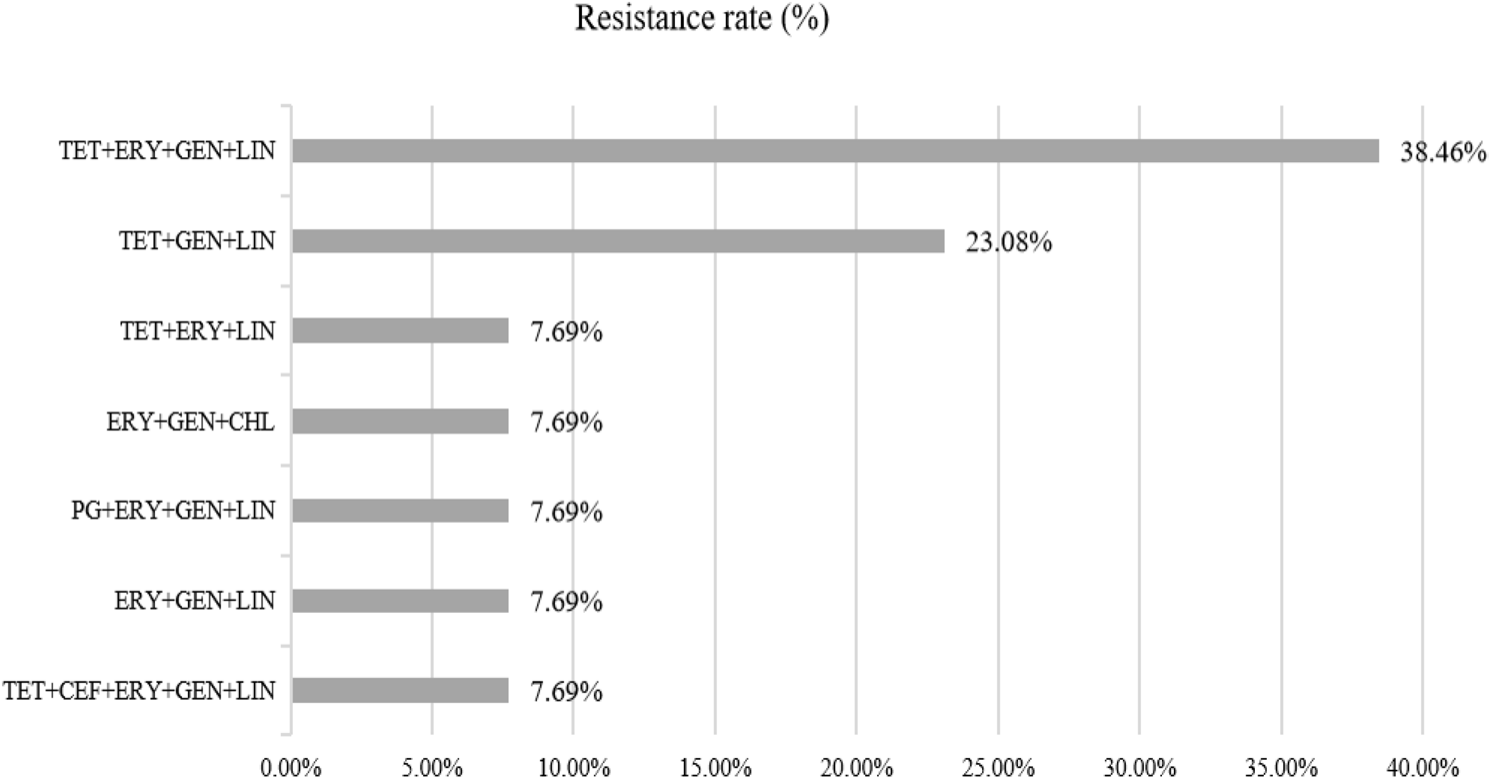
The resistance spectrum of *C. perfringens* strains to various antibiotic combinations. Abbreviations, ERY-Erythromycin, GEN-Gentamicin, LIN-Lincomycin, TET-Tetracycline.PG- Penicillin, CEF- Ceftriaxone

The most common resistance phenotypes observed in the *C. perfringens* isolates were against gentamycin (89.47%), followed by tetracycline (31.58%), lincomycin (29.82%), and erythromycin (22.80%), as detailed in the Table 2. It is worth noting that all the isolates tested showed susceptibility to piperacillin and meropenem. And other antibiotics also exhibited relatively high sensitivity including ampicillin (92.98%), chloramphenicol (94.73%), levofloxacin (98.25%), ceftriaxone (94.73%), and penicillin (98.25%). Among the *C. perfringens* isolates tested, 22.80% (13/57) were classified as multidrug-resistant strains, which were resistant to three or more antibiotics. Additionally, 14.03% (8/57) of the isolates were resistant to four commonly used antibiotics, and one isolate was even resistant to five antibiotics. (Figures 1 and 3).

**Table 2:** MICs distributions of 57 *C. perfringens* isolates against 11 antimicrobial agents

Antimicrobial agents	Number of isolates for different MICs (µg/mL)												Number of Resistant isolates (%)
	≤ 0.0625	0.125	0.25	0.5	1	2	4	8	16	32	64	≥ 128	
Meropenem	54	1			1		1						0
Tetracycline	13	3		2	1	4	8	8	9	6	3		18 (31.58)
Ceftriaxone	42	1	1	1	6	1	1	1		2		1	1 (1.75)
Levofloxacin	10	15	21	8	1	1	1						2 (3.50)
Piperacillin	46	1	5	3	2								0
Penicillin	51	1	2	2	1								0
Ampicillin	48	4		1	1	2					1		3(5.26)
Erythromycin	16	1	1	2	5	13	6			1		12	13 (22.80)
Gentamycin	2		1		1		1	1	3	7	13	28	51 (89.47)
Chloramphenicol	3	1		2	3	15	22	10		1			1 (1.75)
Lincomycin	4	3	2	13	3	7	8	8	1	2		6	17 (29.82)

The shaded fields denote the number of the resistant isolates

### MLST analysis

**Fig. 2:**
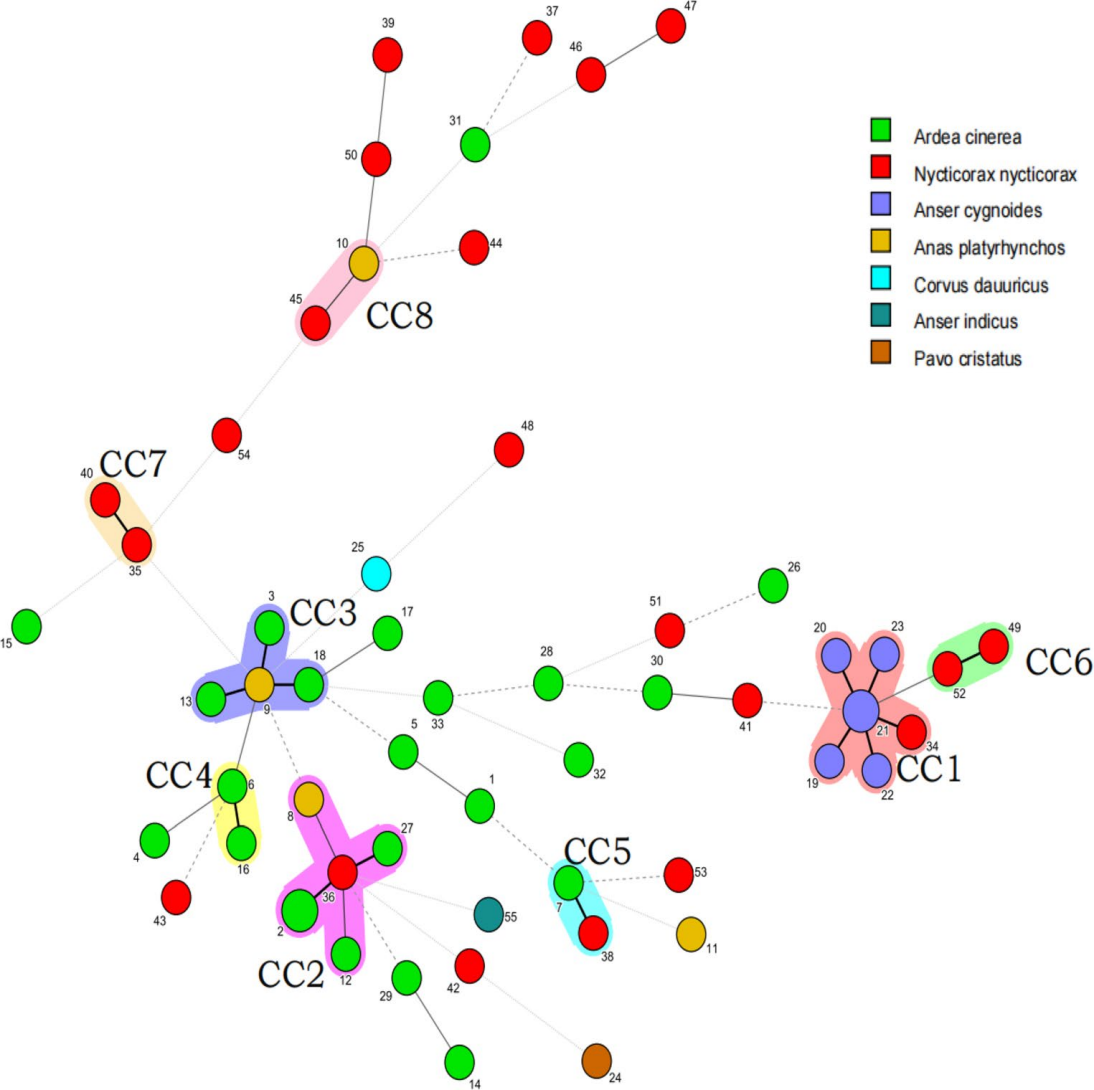
A total of 57 strains of *C. perfringens* from different sources were analyzed by constructing an MLST-minimal spanning tree. The minimum spanning tree was constructed using the Bionumerics software (Bionumerics, version 7.0). The shaded section represents eight clone complexes. The number of the circle represents the sequence type; different colors represent different hosts

MLST result showed that the average number of alleles for all analyzed loci was 20.1. As shown in Fig. 2, the *ddla* gene exhibited the highest level of polymorphism with 40 alleles, while the *gmk* gene had the lowest level of polymorphism with only 10 alleles. In total, 57 isolates of *C. perfringens* were analyzed and classified into 55 STs, of which 23 isolates from grey heron were divided into 22 STs and 21 isolates from night heron were divided into 21 STs. Among these, ST2 included two strains isolated from grey herons, ST21 contained two strains isolated from swan geese, and the remaining 53 STs each consisted of a single strain. The Ds of all isolates in ST was 0.9812, indicating a high level of genetic diversity within *C. perfringens* from wild birds.

**Fig. 3:**
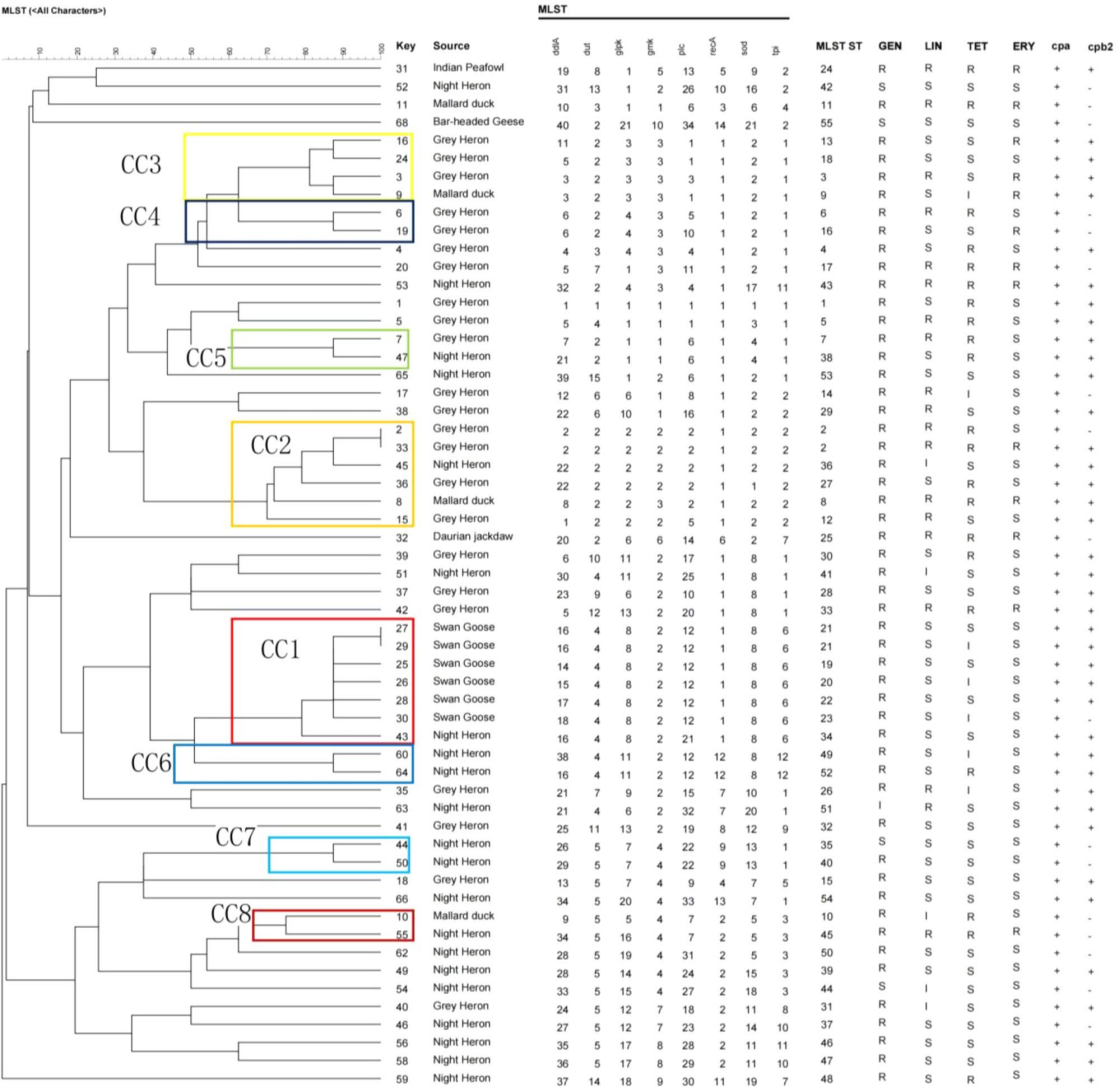
Phylogenetic tree and allelic profiles of 57 *Clostridium perfringens* sequence types (STs)

The minimum spanning tree mainly consisted of eight clonal complexes (CC1-CC8), accounting for 43.9% (25/57) of the examined isolates from different birds. As is shown in Fig. 3, CC1 was the largest CC, contained 7 (12.3%, 7/57) strains (ST19, ST20, ST21, ST22, ST23 and ST34). All the strains isolated from swan goose were clustered in CC1, and ST34 isolated from night heron was also clustered in CC1. CC2 contained 6 (10.5%, 6/57) strains (ST2, ST8, ST12, ST27, ST36) from grey heron (*n* = 4), night heron (*n* = 1), and mallard duck (*n* = 1). CC3 contained 4 (7.0% 4/57) strains (ST3, ST9, ST13, ST18) from grey heron (*n* = 3) and mallard duck (*n* = 1), the other CCs (CC4-CC8) only contained 2 strains. Moreover, 32 (56.1%, 32/57) STs were identified as singletons and did not belong to any CC, including the only isolates from Indian peafowl and bar-headed geese.

## Discussion

According to our results, *C. perfringens* was isolated in 17.38% of 328 fecal samples in Beijing, China, which was slightly lower than that from captive wild birds in India (22.5%) [32]. In general, the carriage rates of *C. perfringens* varied significantly among bird species, especially Indian peafowl (3.70%) and Daurian jackdaw (2.32%), which had very low rates. In terms of lifestyle, migratory wild birds had higher carriage rates than non-migratory birds, and aquatic birds had higher rates than terrestrial birds. It is tentatively hypothesized that the variation in the prevalence of *C. perfringens* in different wild birds is due to ecological factors such as feeding habits, habitat preferences and migration patterns. *C. perfringens* was found to be isolated in all seven species of wild birds, suggesting that wild birds could potentially serve as a reservoir for this bacterium. All the isolates were genotyped for the toxins, and the type A separation rate is 100%, which is in accordance with earlier reports regarding the global dominance of type A [33–36]. Furthermore, the β2 toxin was accounting for 70.18% of all the isolates, which are encoded by the *cpb2* gene and can be produced by all types of *C. perfringens*. There is currently no clear consensus on the pathogenicity of the β2 toxin and its role in disease, as it may be associated with various diseases. It has been found that the expression level of the cpb2 gene is significantly higher in children with autism compared to normal children [37, 38]. Bueschel et al. found the majority of isolates from cases of porcine enteritis and porcine neonatal enteritis were 85% and 91.8% positive for *cpb2* gene, respectively [39].

Previous studies have shown that antibiotic resistance of *C. perfringens* varies significantly between different countries. In Egypt, 100% of the strains of *C. perfringens* were found to be resistant to lincomycin, which is significantly higher than the strains in this study (29.82%) [40]. The resistance rate of the *C. perfringens* strains to tetracycline observed in this study (31.58%) was found to be similar to that reported in India (27.5%) but much lower than the rates observed in Korea (100%) [32, 41]. *C. perfringens* strains have exhibited high resistance to gentamicin in many studies [29, 40], with the resistance levels approaching nearly 100%, and high resistance rates were also observed in the present study (89.47%). Moreover, treatment of *C. perfringens* from equine with gentamicin or streptomycin could induce expression of the β2 toxin and lead to a more accentuated and fatal progression of equine typhlocolitis [42]. β-lactams, as an antibacterial with strong susceptibility to *C. perfringens*, play an important role in the treatment of *C. perfringens* disease in animals [43]. More than 90% of the isolates in this experiment were sensitive to all three β-lactam antibiotics. Considering the rising rate of antimicrobial resistance, there is a need for continuous monitoring of the antimicrobial susceptibility of *C. perfringens* in wild birds to minimize resistance trends for effective prevention and treatment of associated diseases.

The prevalence of multi-resistant strains in wild birds (22.80%) was relatively lower compared to those isolated from animals [29–31], meat products [20] and human clinics [10], probably due to the extensive use of antibiotics in animal husbandry and human healthcare, whereas wild birds have limited direct exposure to antibiotics, resulting in less antibiotic pressure on the bacteria they harbor. Even though the prevalence of multi-resistant strains may be lower in wild birds, the potential risk of wild birds carrying drug-resistant bacteria should not be overlooked.

MLST is usually used for comparing the genetic evolution of *C. perfringens* in animals, and the housekeeping genes used for MLST analysis of *C. perfringens* varied from study to study. In addition to the two groups of housekeeping genes described in the introduction, Hibberd used eight housekeeping genes, *ddl*, *dnaK*, *glpK*, *recA*, *gyrA*, *groEL*, *tpi* and *plc* for MLST analysis of the strain [28], and we were unable to compare them with the strains in their studies, which is a shortcoming of this typing method. In order to better analyze the genetic diversity of the strains carried by wild birds, we compared them to strains from studies that used the same housekeeping genes. In our study, 57 isolates from different wild birds were composed of 55 STs, 7 CCs and the Ds is 0.9812. Xu et analyzed 74 strains of *C. perfringens* from chicken and found an average of 29 alleles, 65 STs, 11 CCs and the Ds is 0.9799 [31]. Li et analyzed 85 strains of *C. perfringens* from duck and encompassed 54 STs, 5 CCs and the Ds is 0.9556 [12]. By comparing the Simpson Diversity Index, the diversity of sequence types of wild birds was obviously higher than the strain isolated from other poultry. The reason for this maybe that wild birds have a greater range and can be infected by *C. perfringens* through contact with different animals or objects compared to the intensive breeding of poultry, and therefore have a higher genetic diversity.

The present study found that CC1 includes all the strains isolated from swan geese, indicating a relatively conserved genetic diversity within the swan goose population. The proximity and social interactions within flocks create a conducive environment for the exchange of bacteria among individuals, potentially leading to the spread and maintenance of specific genetic lineages within the swan goose community. The isolates from grey heron and night heron were scattered in different CCs, the sole isolate of mallard duck and a strain isolated from night heron formed the CC8, while neither the strains from the black swan nor the blue peacock participated in any of the CCs. These results indicated that strains of *C. perfringens* from wild birds exhibited a high degree of genetic variability across different host species, reflecting the complexity of transmission dynamics and evolutionary pressures within different ecological niches. In addition, the cpb2 gene was widely distributed among *C. perfringens* populations in diverse bird hosts without specific relatedness. The limitations of the present study were that only one strain of *C. perfringens* was isolated from bar-headed geese, Indian peafowl and Daurian jackdaw, and these few strains of *C. perfringens* were not sufficient to represent all the strains from these birds.

## Conclusion

In summary, this is the first study that reported the prevalence, toxin type, antimicrobial resistance and genetic diversity of *C. perfringens* from wild birds in Beijing, China. The result indicates that *C. perfringens* exhibits a wide range of host adaptations, varying degrees of antimicrobial resistance, and a high degree of genetic diversity in wild birds. However, further studies are needed to link the occurrence of *C. perfringens* in wild birds to human *C. perfringens* cases and transmission to other animals.

## Data Availability

The datasets used and analysed during the current study are included in the article.

## References

[1] Uzal FA, Freedman JC, Shrestha A, Theoret JR, Garcia J, Awad MM (2014). Towards an understanding of the role of *Clostridium perfringens* toxins in human and animal disease. Future Microbiol.

[2] Immerseel F, Van BJ, De, Pasmans F, Huyghebaert G, Haesebrouck F, Ducatelle R (2004). *Clostridium perfringens* in poultry: an emerging threat for animal and public health. Avian Pathol.

[3] Rood JI, Adams V, Lacey J, Lyras D, McClane BA, Melville SB (2018). Expansion of the *Clostridium perfringens* toxin-based typing scheme. Anaerobe.

[4] Mehdizadeh Gohari I, Navarro A, Li M, Shrestha J, Uzal A (2021). A. McClane B. Pathogenicity and virulence of *Clostridium perfringens*. Virulence.

[5] Li J, Adams V, Bannam TL, Miyamoto K, Garcia JP, Uzal FA (2013). Toxin plasmids of *Clostridium perfringens*. Microbiol Mol Biol Rev.

[6] Freedman JC, Theoret JR, Wisniewski JA, Uzal FA, Rood JI, McClane BA (2015). *Clostridium perfringens* type A-E toxin plasmids. Res Microbiol.

[7] Quainoo S, Coolen JPM, van Hijum SAFT, Huynen MA, Melchers WJG, van Schaik W (2017). Whole-genome sequencing of bacterial pathogens: the future of nosocomial outbreak analysis. Clin Microbiol Rev.

[8] Jost BH, Trinh HT, Songer JG (2006). Clonal relationships among *Clostridium perfringens* of porcine origin as determined by multilocus sequence typing. Vet Microbiol.

[9] Adams V, Han X, Lyras D, Rood JI (2018). Antibiotic resistance plasmids and mobile genetic elements of *Clostridium perfringens*. Plasmid.

[10] Kiu R, Hall LJ (2018). An update on the human and animal enteric pathogen *Clostridium perfringens*. Emerg Microbes Infect.

[11] Mwangi S, Timmons J, Fitz-coy S, Parveen S (2019). Characterization of *Clostridium perfringens* recovered from broiler chicken affected by necrotic enteritis. Poult Sci.

[12] Liu Y, Xiu L, Miao Z, Wang H (2020). Occurrence and multilocus sequence typing of *Clostridium perfringens* isolated from retail duck products in Tai’an region, China. Anaerobe.

[13] Gharaibeh S, Al Rifai R, Al-Majali A (2010). Molecular typing and antimicrobial susceptibility of *Clostridium perfringens* from broiler chickens. Anaerobe.

[14] Chalmers G, Martin SW, Hunter DB, Prescott JF, Weber LJ, Boerlin P (2008). Genetic diversity of *Clostridium perfringens* isolated from healthy broiler chickens at a commercial farm. Vet Microbiol.

[15] Wang Y, Lv Y, Cai J, Schwarz S, Cui L, Hu Z (2015). A novel gene, *optrA*, that confers transferable resistance to oxazolidinones and phenicols and its presence in *Enterococcus faecalis* and *Enterococcus faecium* of human and animal origin. J Antimicrob Chemother.

[16] Du J, Luo J, Huang J, Wang C, Li M, Wang B (2019). Emergence of genetic diversity and multi-drug resistant *Campylobacter jejuni* from wild birds in Beijing, China. Front Microbiol.

[17] Aul P, Ey DF, Afranek HJS, Upp AER, Unne IFD, Wen ECI (2000). Ceftriaxone-resistant *Salmonella* infection acquired by a child from cattle. N Engl J Med.

[18] Craven SE, Stern NJ, Line E, Bailey JS, Cox NA, Fedorka-Cray P (2000). Determination of the incidence of *Salmonella* spp., *Campylobacter jejuni*, and *Clostridium perfringens* in wild birds near broiler chicken houses by sampling intestinal droppings. Avian Dis.

[19] Landers TF, Cohen B, Wittum TE, Larson EL (2012). A review of antibiotic use in food animals: perspective, policy, and potential. Public Health Rep.

[20] Beres C, Colobatiu L, Tabaran A, Mihaiu R, Mihaiu M (2023). Prevalence and characterization of *Clostridium perfringens* isolates in Food-Producing animals in Romania. Microorganisms.

[21] Anju K, Karthik K, Divya V, Mala Priyadharshini ML, Sharma RK, Manoharan S (2021). Toxinotyping and molecular characterization of antimicrobial resistance in *Clostridium perfringens* isolated from different sources of livestock and poultry. Anaerobe.

[22] Yadav JP, Das SC, Dhaka P, Vijay D, Kumar M, Mukhopadhyay AK (2017). Molecular characterization and antimicrobial resistance profile of *Clostridium perfringens* type a isolates from humans, animals, fish and their environment. Anaerobe.

[23] Yadav JP, Das SC, Dhaka P, Mukhopadhyay AK, Chowdhury G, Naskar S (2018). Pulsed-field gel electrophoresis of enterotoxic *Clostridium perfringens* type a isolates recovered from humans and animals in Kolkata, India. Int J Vet Sci Med.

[24] Baums CG, Schotte U, Amtsberg G, Goethe R (2004). Diagnostic multiplex PCR for toxin genotyping of *Clostridium perfringens* isolates. Vet Microbiol.

[25] Bailey MA, Macklin KS, Krehling JT (2013). Use of a Multiplex PCR for the detection of toxin-encoding genes *netB* and *tpeL* in strains of *Clostridium perfringens*. ISRN Vet Sci.

[26] Xiao X, Zhang Q, Wu S, Li Y, Zhong Z, Guo Q (2023). Detection of *Clostridium perfringens* using novel methods based on recombinase-aided amplification assay-assisted CRISPR/Cas12a system. Transbound Emerg Dis.

[27] Weinstein MP, Lewis JS (2020). The Clinical and Laboratory Standards Institute Subcommittee on Antimicrobial susceptibility testing: background, organization, functions, and processes. J Clin Microbiol.

[28] Hibberd MC, Neumann AP, Rehberger TG, Siragusa GR (2011). Multilocus sequence typing subtypes of poultry *Clostridium perfringens* isolates demonstrate disease niche partitioning. J Clin Microbiol.

[29] Xiu L, Liu Y, Wu W, Chen S, Zhong Z, Wang H (2020). Prevalence and multilocus sequence typing of *Clostridium perfringens* isolated from 4 duck farms in Shandong province, China. Poult Sci.

[30] Mehmood K, Li K, Huang SC, Zhang S, Suolang S (2022). Antimicrobial susceptibility and multilocus sequence typing of *Clostridium perfringens* isolated from yaks in Qinghai-Tibet plateau, China. Front Vet Sci.

[31] Xu W, Wang H, Liu L, Miao Z, Huo Y, Zhong Z (2021). Prevalence and characterization of *Clostridium perfringens* isolated from different chicken farms in China. Anaerobe.

[32] Milton AAP, Sanjukta R, Gogoi AP, Momin KM, Priya GB, Das S (2020). Prevalence, molecular typing and antibiotic resistance of *Clostridium perfringens* in free range ducks in Northeast India. Anaerobe.

[33] Azimirad M, Gholami F, Yadegar A, Knight DR, Shamloei S, Aghdaei HA (2019). Prevalence and characterization of *Clostridium perfringens* toxinotypes among patients with antibiotic-associated diarrhea in Iran. Sci Rep.

[34] Fayez M, Elsohaby I, Al-Marri T, Zidan K, Aldoweriej A, El-Sergany E (2020). Genotyping and antimicrobial susceptibility of *Clostridium perfringens* isolated from dromedary camels, pastures and herders. Comp Immunol Microbiol Infect Dis.

[35] Verma AK, Abdel-Glil MY, Madesh A, Gupta S, Karunakaran AC, Inbaraj S (2020). Multilocus sequence typing of *Clostridium perfringens* strains from neonatal calves, dairy workers and associated environment in India. Anaerobe.

[36] Xu W, Zhang H, Hu Z, Miao Z, Zhang Y, Wang H (2021). Prevalence and multilocus sequence typing of *Clostridium perfringens* isolated from retail chicken products and diseased chickens in Tai’an region, China. Vet Med Sci.

[37] Finegold SM, Summanen PH, Downes J, Corbett K, Komoriya T (2017). Detection of *Clostridium perfringens* toxin genes in the gut microbiota of autistic children. Anaerobe.

[38] Góra B, Gofron Z, Grosiak M, Aptekorz M, Kazek B, Kocelak P (2018). Toxin profile of fecal *Clostridium perfringens* strains isolated from children with autism spectrum disorders. Anaerobe.

[39] Bueschel DM, Jost BH, Billington SJ, Trinh HT, Songer JG (2003). Prevalence of cpb2, encoding beta2 toxin, in *Clostridium perfringens* field isolates: correlation of genotype with phenotype. Vet Microbiol.

[40] Hamza D, Dorgham S, Hakim A (2017). Toxinotyping and antimicrobial resistance of *Clostridium perfringens* isolated from processed chicken meat products. J Vet Res.

[41] Jang YS, Kim DH, Bae D, Kim SH, Kim H, Moon JS (2020). Prevalence, toxin-typing, and antimicrobial susceptibility of *Clostridium perfringens* from retail meats in Seoul, Korea. Anaerobe.

[42] Vilei EM, Schlatter Y, Perreten V, Straub R, Popoff MR, Gibert M (2005). Antibiotic-induced expression of a cryptic *cpb2* gene in equine β2-toxigenic *Clostridium perfringens*. Mol Microbiol.

[43] Johansson A, Greko C, Engström BE, Karlsson M (2004). Antimicrobial susceptibility of Swedish, Norwegian and Danish isolates of *Clostridium perfringens* from poultry, and distribution of tetracycline resistance genes. Vet Microbiol.

